# Effect of vitamin D supplementation on N‐glycan branching and cellular immunophenotypes in MS

**DOI:** 10.1002/acn3.51148

**Published:** 2020-08-23

**Authors:** Priscilla Bäcker‐Koduah, Carmen Infante‐Duarte, Federico Ivaldi, Antonio Uccelli, Judith Bellmann‐Strobl, Klaus‐Dieter Wernecke, Michael Sy, Michael Demetriou, Jan Dörr, Friedemann Paul, Alexander Ulrich Brandt

**Affiliations:** ^1^ Charité – Universitätsmedizin Berlin, Corporate member of Freie Universität Berlin, Humboldt – Universität zu Berlin, and Berlin Institute of Health, NeuroCure Cluster of Excellence Berlin Germany; ^2^ Experimental and Clinical Research Center Max Delbrueck Center for Molecular Medicine and Charité – Universitätsmedizin Berlin Berlin Germany; ^3^ Charité ‐ Universitätsmedizin Berlin Institute for Medical Immunology Berlin Germany; ^4^ Department of Neuroscience, Rehabilitation, Ophthalmology, Genetics, Maternal and Child Health CEBR University of Genoa Genoa Italy; ^5^ Ospedale Policlinico San Martino‐IRCCS Genoa Italy; ^6^ Berlin Institute of Health NeuroCure Cluster of Excellence Department of Neurology Charité – Universitätsmedizin Berlin Berlin Germany; ^7^ Institute of Biometry and Clinical Epidemiology Charité ‐Universitatsmedizin Berlin and CRO SOSTANA GmbH Berlin Germany; ^8^ Department of Neurology University of California Irvine CA USA; ^9^ Multiple Sclerosis Center Hennigsdorf Oberhavel Clinics Berlin Germany

## Abstract

**Objective:**

To investigate the effect of cholecalciferol (vitamin D3) supplementation on peripheral immune cell frequency and N‐glycan branching in patients with relapsing‐remitting multiple sclerosis (RRMS).

**Methods:**

Exploratory analysis of high‐dose (20 400 IU) and low‐dose (400 IU) vitamin D3 supplementation taken every other day of an 18‐month randomized controlled clinical trial including 38 RRMS patients on stable immunomodulatory therapy (NCT01440062). We investigated cholecalciferol treatment effects on N‐glycan branching using L‐PHA stain (*phaseolus vulgaris leukoagglutinin*) at 6 months and frequencies of T‐, B‐, and NK‐cell subpopulations at 12 months with flow cytometry.

**Results:**

High‐dose supplementation did not change CD3+ T cell subsets, CD19+ B cells subsets, and NK cells frequencies, except for CD8+ T regulatory cells, which were reduced in the low‐dose arm compared to the high‐dose arm at 12 months. High‐dose supplementation decreased N‐glycan branching on T and NK cells, measured as L‐PHA mean fluorescence intensity (MFI). A reduction of N‐glycan branching in B cells was not significant. In contrast, low‐dose supplementation did not affect N‐glycan branching. Changes in N‐glycan branching did not correlate with cell frequencies.

**Interpretation:**

Immunomodulatory effect of vitamin D may involve regulation of N‐glycan branching *in vivo*. Vitamin D3 supplementation did at large not affect the frequencies of peripheral immune cells.

## Introduction

Multiple sclerosis (MS) is considered a T‐cell‐mediated disease, but other immune cells have been implicated in its pathology, most notably B and NK cells.^1^, ^2^, ^3^ Characteristics are a disruption of T, B, and NK regulatory cells^4^, ^5^, ^6^ with reduced levels of T regulatory cells (Tregs),^7^ and impaired B regulatory cells (Bregs) function.^8^ Genetic and environmental factors contribute to risk, age at onset, and progression of MS.^9^, ^10^, ^11^ Among the latter are sunlight exposure and 25‐hydroxyvitamin D (25(OH)D).^9^, ^10^, ^12^, ^13^, ^14^, ^15^, ^16^, ^17^ Epidemiological, retrospective, and a few interventional studies have investigated vitamin D in MS. But larger interventional studies are scarce^18^, ^19^ and are increasingly difficult to conduct as patients self‐supplement vitamin D.

Exact mechanisms by which vitamin D influences MS and its clinical efficacy when supplemented remain to be elucidated. Animal studies and *in vitro* assays proposed an immunomodulatory role of 25(OH)D.^20^, ^21^, ^22^ *In vitro*, the active metabolite, 1,25(OH)2D3 downregulates CD4+ T cell interleukin‐17 (IL‐17) while upregulating interleukin‐10 (IL‐10)^23^ production. Furthermore, 1,25 (OH)2D3 inhibits antibody production by plasma cells^22^ and promotes differentiation of CD4+ T cells into immunomodulatory T helper 2 (Th2) cells.^21^ Human *in vivo* studies investigating 25(OH)D in MS have produced varying results regarding its effect on proinflammatory and anti‐inflammatory factors.^24^, ^25^ A recent randomized controlled trial in 40 RRMS patients showed a dose‐dependent reduction in the proportion of IL‐17+ CD4+ T cells.^19^

One known effect how vitamin D regulates immune cells is through effects on cell‐surface protein glycosylation, that is, by regulation of enzymes of the glycosylation pathway.^11^, ^26^ Cell‐surface proteins are co and posttranslationally modified by N‐glycosylation, a process that adds sugars to proteins on asparagine (N) residues through N‐glycosidic bonds (N‐glycans).^27^, ^28^, ^29^ Complex N‐glycans are branched via the addition of N‐acetyl‐D‐glucosamine (GlcNAc) by N‐acetylglucosaminyltransferases (Mgat) enzymes in the Golgi apparatus.^29^, ^30^ Four branching enzymes act sequentially with declining efficiency to generate N‐glycans with up to four GlcNAc branches, namely Mgat 1, 2, 4, and 5.^31^ Upregulation or downregulation of Mgat 1 activity reduces N‐glycan branching by limiting the substrate, uridine diphosphate N‐acetylglucosamine, UDP‐GlcNAc.^11^ Interestingly, vitamin D seems to upregulate N‐glycan branching, as exposure of activated mouse or human T cells to 1,25(OH)2D3 raises N‐glycan branching by increasing Mgat1 mRNA, whereas it lowers N‐glycan branching in deficient mice.^11^ Hence, 1,25(OH)D2D3 and UDP‐GlcNAc availability determine N‐glycan synthesis by Mgat enzymes.^11^, ^32^

Reduced N‐glycan branching lowers T‐cell activation threshold, drives proinflammatory Th1 and Th17 differentiation, and inhibits anti‐inflammatory Treg responses.^29^, ^33^, ^34^ Conversely, T‐cell activation increases N‐glycan branching in T cells blasts, which serve as negative feedback to terminate T‐cell responses.^33^, ^35^ On the other hand, genetic variations in IL‐2R and IL‐7R signaling increases the risk of MS by reducing branching on T‐cell blasts.^11^, ^32^ In mouse models, reducing N‐glycan branching promotes spontaneous autoimmunity including inflammatory demyelination,^28^, ^29^, ^36^ whereas raising branching attenuates immune responses.^37^, ^38^

Thus, activated or partially activated T cells could hypothetically be suppressed by vitamin D supplementation in MS via increase in N‐glycan branching. But human interventional studies investigating the effect of vitamin D supplementation on N‐glycan branching have not been performed to date, leaving it unclear if this mechanism is of actual physiological relevance *in vivo* in humans.

In the ‘Efficacy of Vitamin D supplementation In Multiple Sclerosis’ (EVIDIMS) trial, (NCT01440062) patients with RRMS received either high‐dose (20 400 IU) or low‐dose (400 IU) cholecalciferol every other day over 18 month.^39^ Patients were monitored clinically and with brain magnetic resonance imaging (MRI).^39^, ^40^ The primary endpoint of cumulative new T2w hyperintense lesions was missed with no serious adverse event.^40^ A high prevalence of 25(OH)D deficiency was recorded at baseline and this was associated with increased disease activity prior to supplementation.^41^ In this exploratory substudy, we investigated the immunomodulatory properties of vitamin D3 on (a) frequencies of immune cells associated with MS pathology and (b) N‐glycan branching on immune cells.

## Materials and Methods

### Subjects

Detailed inclusion/exclusion criteria have been published elsewhere.^39^, ^40^ In brief, patients with RRMS according to the 2005 McDonald criteria,^42^ 30 days of no relapse before study entry, age of 18‐65 years, EDSS score between 0.0 and 6.0 were included. Patients were on stable IFNβ‐1b treatment at least 3 months before study entry. Exclusion criteria were the presence of other autoimmune diseases or immunomodulatory therapy besides IFNβ‐1b.

Patients were recruited from a single region in north‐eastern Germany and were randomized in 1:1 to either high or low‐dose arm stratified according to gender and serum 25(OH)D levels (< or ≥20 ng/mL (50 nM)) at screening.^40^ Patients were examined at baseline, 3, 6, 12, and 18 months, and at each visit blood samples were collected for serum and peripheral blood mononuclear cells (PBMCs).

In this substudy, we included 38 patients (Table 1). Patients were excluded if they violated the study protocol: lost to follow‐up, change in MS medication, personal reasons, MRI no longer performable, psychological problems, or if their serum 25(OH)D levels were below 2× the baseline mean compared to the reference level at 6 months for the high‐dose arm and vice versa for the low‐dose arm. Two patients from the high‐dose arm were excluded because serum 25(OH)D levels indicated nonresponse or noncompliance according to this rule.^40^ The trial successfully raised 25(OH)D serum levels with a significant increase in the high‐dose arm compared to the low‐dose arm (p < 0.001) (Fig. S1), supporting the validity of this exploratory analysis.

**Table 1:** Baseline Demographics and univariate analysis of clinical parameters at 6 and 12 months.

Variable	Experiment 1 (n = 29)						Experiment 2 (n = 38)					
	Baseline			12 months			Baseline			6 months		
	High dose	Low dose	*P* value	High dose	Low dose	*P* value	High dose	Low dose	*P* value	High dose	Low dose	*P* value
Male n (%)	6(54.5)	5 (45.4)	0.999^1^				6 (46.2)	7 (53.8)				
Female n (%)							15 (60.0)	10 (40.0)	0.502^1^			
Age in years							41.2 (12.1)	43.1 (9.6)	0.527^2^			
mean (SD)												
Serum 25(OH)	20 (11)	17 (7)	0.678^2^	76 (18)	23 (5)	<0.001^2^	18.7(9.7)	16.5 (8.1)	0.428^2^	73.9 (18.4)	23.3 (4.81)	<0.001^2^
mean (SD) ng/mL												
EDSS, median [interquartile range]	2 [1.2]	2.5 [2.0]	0.112^2^	2 [1.0]	2.5 [2.4]	0.261^2^	2.3 [0.6]	2.0 [2.0]	0.634^2^	2.5 [1.1]	2.3 [2.1]	0.711^2^

^1^ Exact χ^2^‐test.

^2^ Exact Mann–Whitney‐test.

### Ethics approval

This study is an ancillary study of the EVIDIMS trial (NCT01440062), a German multicenter, stratified, randomized, controlled, and double‐blind clinical phase II pilot study. The study was approved by the German Federal Institute for Drugs and Medical Devices and the Ethics Committee of the State of Berlin at the Office for Health and Social Affairs. All patients gave written informed consent.

### Serum 25(OH)D measurement

Serum 25(OH)D levels were measured by Bioscientia Institute for Medical Diagnostics, GmbH Berlin, Germany, using the LIAISON® Fa. DiaSorin chemiluminescence analyzer (DiaSorin, Dietzenbach, Germany).

### Isolation of PBMCs

PBMCs were isolated using 10 mL of Biocoll separating solution (Biochrom GmbH, Darmstadt, Germany) and 10 mL blood by gradient centrifugation. Aliquots of 10 million cells were frozen in liquid nitrogen with cryoprotection until immunological assays were performed.

Investigators were blinded to the different treatment arms during the conduction of the experiments and analysis until after the first preliminary data analyses.

### Immunophenotyping for frequencies of effector and regulatory immune cells

We followed the protocol and antibody panel as previously published by the Sys4MS (Systems medicine approach For MS) study.^43^ The Sys4MS study is a European consortium, which aims to personalize healthcare in MS using a systems medicine approach.

Briefly, three customized single‐batch lyotubes (BD, USA) with antibody cocktails were used for identifying, T effector cells (Teff), Treg, B, and NK effector/regulatory (B/NK) cells.

The B/NK lyotubes contained antibodies for staining CD19, CD3, CD24, CD16, CD38, CD14, CD56, the Treg lyotubes contained antibodies for staining CD3, CD4, CD8, CD28, CD25, CD45RA, CD127, and the Teff lyotubes for staining, CD3, CD4, CD8, CD161, CXCR3, CCRA, and CCR6.

Frozen PBMCs were thawed in a water bath at 37°C and suspended in 2 mL Ca2+‐ and Mg2+‐free PBS. Cells were washed and resuspended in 5 mL cold FACS buffer (PBS + 1% Fetal calf serum, FCS) and counted. The pellets were resuspended in FACS buffer to 1 million cells /100 µL.

Lyotubes were rehydrated using 100 µL of Brilliant stain buffer (BD, Cat. #563794) for 5 minutes at 4°C and 1 million PBMCs added to each lyotube.

PBMCs and antibodies were incubated for 30 minutes at 4°C and washed. Dead cells were identified using 500 µL of Fixable viable stain, FVS 520 (2.5 µL in 50 mL PBS) (BD, Cat. #564407) for 10 minutes at 4°C and washed. Cells were acquired same day without fixation using the FACSCanto II with Diva 6.1.3.8 (BD Pharmingen, Franklin Lakes, NJ, USA).

Storage events were set to the immune cell population of interest, 15,000 events were recorded for the Treg tubes and Teff tubes and 5000 for the B/NK tubes.

### Flow cytometric analysis for β1,6 N‐glycan expression on immune cells

A cocktail of antibodies for L‐PHA‐FITC (2 µg/mL of staining volume) (Vector Laboratories, Cat. #FL‐1111), CD45 PerCP (1:100, BioLegend, Cat. #368506), CD3 APC (0.5:100, BioLegend, Cat. #317318), CD4 APC‐Cy7 (0.5:100, BioLegend, Cat. #317418), CD8a PB (0.5:100, BioLegend, Cat. #301033), CD19 BV510 (1:100, BioLegend, Cat. #302242), CD16 APC/Cy7 (0.5:100, BioLegend, Cat. #302018), CD56 PB (0.5:100, BioLegend, Cat. #362520) was prepared in PBS (‐ Ca+ and ‐ Mg+).

PBMCs (1 × 10^6^ cells) were incubated for 30 minutes at 4°C with the antibody cocktail after Fc blockade (0.25 mg BD, Cat: #564220). Cells were resuspended in 500 µL FACS buffer and proceeded to acquisition immediately. Cells were stained in duplicates and a total of 20,000 events recorded for each tube. For duplicate acquisitions, the average of the two recorded events was used in the data analysis.

### Statistics

Data distribution was checked using the Shapiro–Wilk test. Results for continuous variables are presented as median [interquartile range] and as mean (SD) for non‐normally and normally distributed data, respectively. For categorical variables, we present data as absolute numbers and relative frequency (%). Immunology data are presented as percentage fold change. Serum 25(OH)D levels over the entire study period were analyzed using a nonparametric multivariate covariance analysis for longitudinal data with baseline as a covariate in a two‐factorial design (1^st^ factor: groups, 2^nd^ factor: time). To compare the changes from baseline to successive visits in the immune cell data, we used a nonparametric analysis of covariance (ANCOVA) for longitudinal data with baseline as a covariate. Univariate comparisons between high‐ and low‐dose arms for particular visits were performed using the nonparametric exact Mann–Whitney test. For experiments with duplicates, the average of the duplicates was used in the analysis. Correlation analyses were performed using Spearman’s Rho. Statistical significance was set as *P* < 0.05. All tests should be understood as constituting exploratory data analysis, such that no adjustments for multiple testing have been made. Data were analyzed in R 3.6.0 (2019‐04‐26) and SAS 9.4 [TS1M3] (SAS Institute Inc., Cary, NC, USA).

## Results

### Vitamin D3 supplementation and immune cell frequency (Experiment 1)

**Figure 1:**
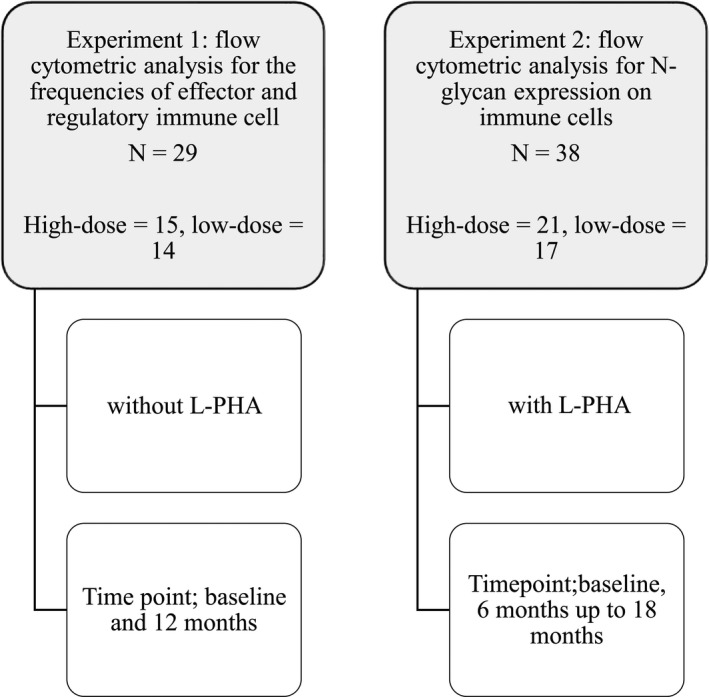
Experimental design flowchart.

To investigate the effect of vitamin D3 supplementation on the frequencies of broad, effector/ regulatory T, B, and NK cells, we compared the percentage fold change at 12 months without L‐PHA stain. The experimental design is illustrated in (Fig. 1).

**Figure 2:**
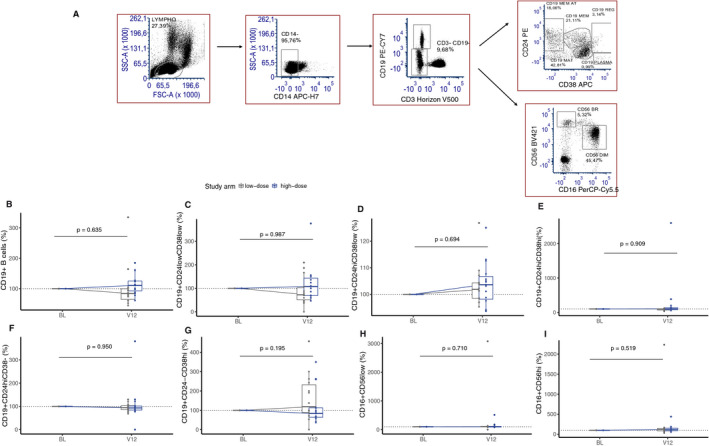
Immune cell frequencies expressed as percentage fold change at 12 months in B and NK cells (B/NK). The figure shows the gating strategy (A) and boxplot of the frequencies of immune cells ( percentage fold change) between high‐dose (blue) and low‐dose (gray) arms in; (B) CD19+ B cells+ (C) CD19+ CD24lowCD38low (B‐mature), (D) CD19+CD24highCD38low (B‐memory), (E) CD19+CD24highCD38high (B‐regulatory), (F) CD19+CD24highCD38‐(B‐memory atypical), (G) CD19+CD24‐CD38high (B‐plasma), (H) CD16+CD56low (CD56dim), and (I) CD16+CD56high (CD56bright). Nonparametric analysis of covariate (ANCOVA) with baseline as covariate (n = 29). Abbreviations: BL, baseline; V12, 12 months.

In group‐wise analyses, high‐ and low‐dose arms did not differ in terms of the frequencies of total CD19+ B cells, B‐mature (CD19+CD24lowCD38low), B‐ memory (CD19+CD24highCD38low), Breg (CD19+CD24highCD38high), B‐memory atypical (CD19+CD24highCD38‐), and B‐plasma (CD19+CD24‐CD38high), cells at 12 months (Fig. 2B–G). High‐dose vitamin D3 did not affect the frequencies of CD56dim cells (CD16+CD56low) and CD56bright cells (CD16+ CD56high) at 12 months (Fig. 2H,I).

**Figure 3:**
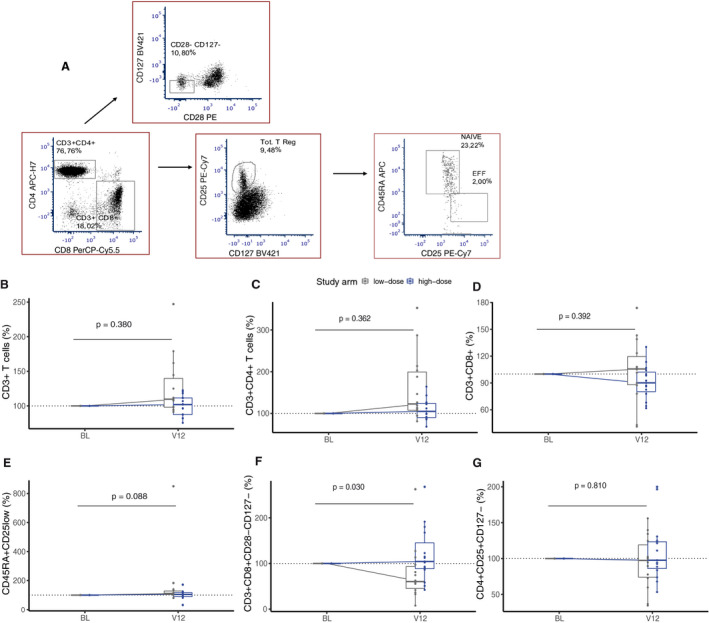
Immune cell frequencies expressed as percentage fold change at 12 months in T regulatory cells (Tregs). The figure shows the gating strategy (A) and boxplot of the frequencies of immune cells (percentage fold change) between high‐dose (blue) and low‐dose (gray) arms in; (B) CD3+ T cells (C) CD3+CD4+ T cells (T helper), (D) CD3+CD8+ T cells (T cytotoxic), (E) CD45RA+CD25low (naïve T regulatory), (F) CD3+CD8+CD28‐CD127‐ (CD8+ T regulatory), and (G) CD3+CD4+CD25+CD127‐ (CD4+ T regulatory). Nonparametric analysis of covariate (ANCOVA) with baseline as covariate (n = 29).

**Figure 4:**
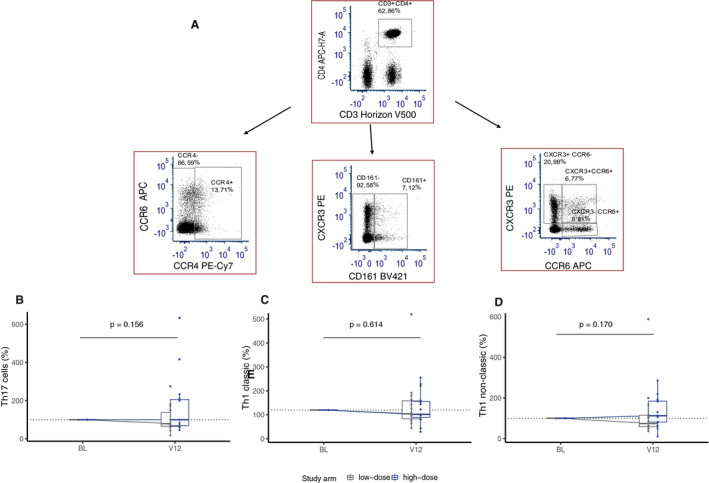
Immune cell frequencies expressed as percentage fold change at 12 months in T effector cells (Teff). Gating strategy (A) and boxplot of immune cell frequencies (percentage fold change) between high‐dose (blue) and low‐dose (gray) arms in; (B) CD3+CD4+CCR6+CD161+CXCR3‐CCR4+ (Th17), (C) CD3+CD4+CCR6‐CD161‐CXCR3+ (Th1), and (D) CD3+CD4+CCR6‐CD161+CXCR3+ (Th1 nonclassic). Nonparametric analysis of covariate (ANCOVA) with baseline as covariate (n = 29).

Likewise, frequencies of CD3+ T cells, CD3+CD4+ T cells, CD3+CD8+ T cells and naïve Tregs (CD45RA + CD25low) did not differ between high‐ and low‐dose arms at 12 months (Fig. 3B–E). In the low‐dose arm, the frequency of CD8+ Tregs (CD8+ CD28‐CD127‐) was reduced compared to the high‐dose arm at 12 months (*P* = 0.030), whereas that of CD4 + Treg (CD4+CD25+CD127‐) cells did not differ between arms at 12 months (Fig. 3F–G). The proportions of Teffs; Th17 cells (CD3+CD4+CCR6+CD161+CXCR3‐CCR4+), Th1 classic (CD3+CD4+CCR6‐CD161‐CXCR3+) and Th1 nonclassic (CD3+CD4+CCR6‐CD161+CXCR3+) cells were not affected by high‐ or low‐dose supplementation at 12 months (Fig. 4B–D).

In analyses combining both arms, we found no correlations between immune cell frequencies and serum 25(OH)D levels at 6, 12, or 18 months (data not shown). An association between CD8+CD28‐CD127‐ and serum 25(OH)D concentration was not confirmed in the correlation analysis (*P* = 0.166).

### Vitamin D3 supplementation and N‐glycosylation (Experiment 2)

We investigated the effect of vitamin D3 supplementation on the intensity of β1,6 N‐glycan expression on broad T, B, and NK cells measured by MFI of L‐PHA, at baseline, 6, 12, and 18 months. L‐PHA binds to β1,6 branched N‐glycans produced by Mgat5 and serves as an overall measure of N‐glycan branching.^29^ The gating strategy is shown in Figure S2.

First, we tested for correlations between MFI of L‐PHA on immune cells and serum 25(OH)D levels at baseline, 6, 12, and 18 months. The time point that showed strong negative correlations of MFI of L‐PHA with serum 25(OH)D levels was 6 months (Fig. S3A‐D). No strong correlations were found at baseline, 12 or 18 months (Fig. S3A‐D). As a result, we focused our analyses at the 6 months’ time point.

**Figure 5:**
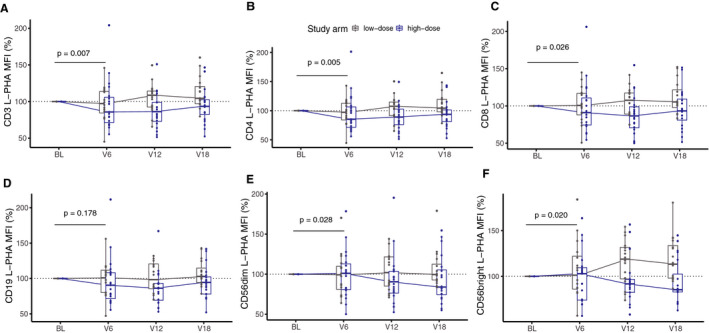
Immune cells L‐PHA MFI over different time points. Boxplots of L‐PHA MFI on immune cells expressed as percentage fold change in T, B, and NK cells. The change in MFI of L‐PHA was compared between baseline and 6 months in the high‐dose arm (blue) and the low‐dose arm (gray) in (A) CD3+ (*P* = 0.007), (B) CD4+ (*P* = 0.005), (C) CD8+ (*P* = 0.026) T cells, (D) CD19+ B cells (*P* = 0.178), (E) CD56bright (*P* = 0.020), and (F) CD56dim, (p = 0.028) NK cells. Nonparametric analysis of covariate (ANCOVA) with baseline as covariate (n = 38). Abbreviations: BL, baseline, V6, 6 months, V12, 12 months, V18, 18 months.

In group‐wise analyses, comparing high‐dose vs. low‐dose arms, high‐dose vitamin D3 supplementation led to a decrease in the MFI of L‐PHA at 6 months on CD3+ (*P* = 0.007), CD4+ (*P* = 0.005), CD8+ (*P* = 0.026) T cells (Fig 5A–C). This decrease lasted up to 18 months compared to the low‐dose arm, which did not change significantly over time. MFI of L‐PHA on CD19+ B cells also showed a reduction although not significant (*P* = 0.178) (Fig. 5D). In NK cells, CD56dim cells showed reduced branching at 6 months in the high‐dose arm (*P* = 0.028) and continuously decreased until 18 months, whereas the low‐dose arm remained stable over time. In CD56bright cells, branching was reduced in the high‐dose arm at 6 months (*P* = 0.020) and decreased steadily until 18 months. The low‐dose arm, on the other hand, continuously increased up to 18 months (Fig. 5E,F).

**Figure 6:**
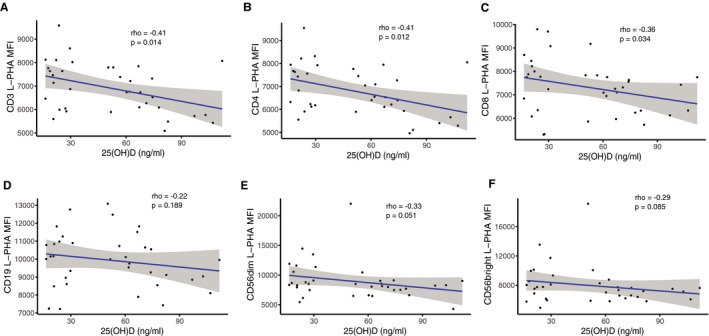
Correlational analyses at 6 months between L‐PHA MFI and serum 25(OH)D levels. Association of serum 25(OH)D levels in both arms with the L‐PHA MFI of (A) CD3+, (B) CD4+, (C) CD8+ T cells, (D) CD19+ B cells, (E) CD56dim, and (F) CD56bright NK cells. Spearman’s Rho (n = 38).

In correlation analyses combining both arms, we found negative correlations between L‐PHA MFI and serum 25(OH)D levels in CD3+, CD4+, CD8+ T cells, CD19+ B cells, CD16 + CD56low (CD56dim) and CD16+CD56high (CD56bright) NK cells. This further confirmed reduced N‐glycan branching intensity observed in the group‐wise analyses (Fig. 6A–F).

### N‐glycan branching and immune cell frequency

**Figure 7:**
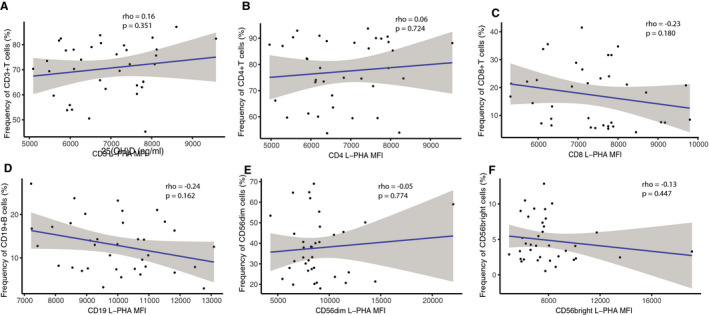
Correlational analyses at 6 months between L‐PHA MFI and the corresponding cell frequencies. The plot of L‐PHA MFI with the corresponding cell frequencies at 6 months in both arms, (A) CD3+, (B) CD4+, (C) CD8+ T cells, (D) CD19+ B cells, (E) CD56dim, and (F) CD56bright NK cells. Spearman’s Rho (n = 38).

To investigate if the observed effect of vitamin D3 on N‐glycan branching was due to differences in immune cell frequency, we tested correlations between cell frequencies and their corresponding L‐PHA MFI. There was no association between cell frequencies and the expression of GlcNAc on CD3+ T cells, CD4+ T cells, CD8+ T cells, CD19+ B cells, CD56dim, and CD56bright NK cells at 6 months (Fig. 7A–F), as well as 12 months or 18 months (data not shown).

## Discussion

Vitamin D3 supplementation did not affect the proportions of Th/Teff cells, Beff/Breg cells, and NK cells subpopulations. Specifically, the proportions of Th1 and Th17, which are known to be disturbed in MS remained stable. We observed a reduction in CD8+ Treg proportions in the low‐dose arm, which remained unchanged in the high‐dose arm. CD8+ Treg is a type of natural Tregs, which lack FoxP3 expression.^44^, ^45^ Similar to CD4+CD25+ Tregs, they induce and maintain peripheral tolerance by regulating harmful and autoreactive T cells.^46^, ^47^ This indicates that high‐dose supplementation may support maintaining CD8+ Treg levels. Although this is an interesting finding, this was not correlated with serum 25(OH)D levels nor with effector T cells raising the likelihood that this exploratory result is false positive.

Vitamin D3 supplementation did not affect naïve Treg proportions (CD45RA+CD25low). This is not surprising as naïve T cells do not express the vitamin D receptor (VDR) and hence respond weakly to the stimulation of the T‐cell receptor by vitamin D.^48^ *In vitro* studies have shown that activated naïve CD4+ T cells cannot convert 25(OH)D to 1,25(OH)D3 when cultured in serum or with vitamin D‐binding protein (DBP).^49^ In contrast, in DBP‐null mice, although 1,25(OH)D3 blood levels were significantly reduced, levels in target tissue were significantly higher compared to wildtype.^50^ This suggests that DBP may have limited impact on distribution, uptake, and biological activity in target tissues.^50^ In comparison, in our study cells were not cultured or prestimulated, hence the effects of DBP or serum on the action of 25(OH)D cannot be directly applied to our results. Moreover, studies that have shown significant effects of vitamin D supplementation in MS, prestimulated immune cells before immunostaining.^19^, ^51^ VDR expression on immune cells is upregulated upon activation,^52^, ^53^, ^54^ and this may explain why studies that prestimulated immune cells before vitamin D treatment show varying results compared to those without prestimulation.

A randomized controlled trial with cholecalciferol supplementation showed no difference in the degree of change from baseline in CD4+CD161+ cells, which are markers for Th17 cells in both high‐ and low‐dose arms. However, a significant decrease was observed in CD4+ IL‐17+ cells proportions in the high‐dose arm.^19^ We did not observe a reduction in the CD161+CD4+ T cells fraction with high‐dose supplementation as reported by Sotirchos et al.,^19^ potentially due to the differences in methodology, time of analysis and supplementation plan. In their, study, patients received additionally 1 000 mg calcium and multivitamins.^19^ Calcium is known to enhance both vitamin D absorption and half‐life of 25(OH)D.^55^ While we investigated the effect of vitamin D after 12 months of supplementation, Sotirchos et al., investigated vitamin D effects after 6 months. In a previous trial, supplementation with 2000 to 8000 IU cholecalciferol daily for 12 weeks reduced frequencies of CD4 + IL‐17 + cells in prestimulated peripheral T cells.^51^ However, no response was observed on B and T cells in a linear‐dose‐dependent manner.^51^

Other studies also could not detect any association or effect between vitamin D and frequencies of Th17, naïve T cells, or Bregs in MS patients.^24^, ^56^, ^57^ The SOLAR study recently reported on a lack of effect of vitamin D on the proportions of Breg and Tregs or on IL‐17 cytokines production between high‐dose and placebo arms.^57^ Similarly, in two randomized placebo‐controlled trials with high‐dose vitamin D supplementation as add‐on to IFNβ, the levels of IL‐17 cytokines were not different between groups.^58^, ^59^ We recently investigated the impact of treatment on immunophenotypes in different subtypes of 227 MS patients under different disease‐modifying therapies (DMT). In this study, we found no changes in frequencies of Th17, Th1, CD4+ Tregs nor Breg/Beff cells in RRMS patients compared to healthy controls. The only DMT that showed differences in lymphocyte populations was Fingolimod, whereas patients on IFN showed no differences in the frequencies of immunophenotypes.^43^

The vitamin D response index; the efficiency with which one responds to vitamin D at the molecular level could also explain the different outcomes on the frequencies of immune cells.^60^ Molecular‐based (gene expression and chromatin accessibility) analyses revealed three types of responders; high, mid, and low responders, implying that individuals may need different amounts of vitamin D to show biologically relevant response.^60^, ^61^, ^62^, ^63^

Our study is the first to investigate N‐glycan branching in the context of vitamin D supplementation trial.

*Ex‐vivo* cells and animal studies have shown that prestimulated human CD4+ T cells treated with 40 ng/ml (100 nM) 1,25(OH)2D3 enhanced Mgat1 mRNA levels with a concurrent increase in N‐glycan branching.^11^ In EAE mice, reduced dietary supplementation decreased N‐glycan branching, whereas injection of 1,25(OH)2D3 inhibited autoimmunity.^11^, ^32^

On the basis of these data, we expected higher N‐glycan branching with high‐dose supplementation, however, we found the opposite effect. In *ex vivo* human PBMCs, high‐dose oral cholecalciferol intake reduced branching in T cells and NK cells. This effect was not dependent on immune cell frequencies, indicating that vitamin D3 downregulated branching via Mgat enzymes.

Resting T cells minimally express the VDR compared to activated T cells,^52^ therefore, activated T cells will primarily respond to vitamin D *in vivo*. Indeed, previous studies showed that 1,25(OH)2D3 increased N‐glycan branching in preactivated, but not resting human/mouse T cells.^33^, ^35^ As most of the patients at enrolment in our study were 25(OH)D deficient; this might have induced a high activation state within the immune system, which might have already increased branching shortly (days to weeks) after supplementation. Thus, vitamin D3 supplementation over time would be expected to lower branching in the activated cells as they revert to a quiescent resting state, with a net effect of decreased branching observed in our study in the high‐dose arm. The effects of vitamin D on N‐glycan branching in activated T cells occur over days *in vitro*,^11^ however, we evaluated changes in branching at 6 months after supplementation. Evaluation at earlier time points would be needed to confirm this hypothesis.

Thus, the effect of vitamin D on N‐glycan branching depends on VDR expression, the activation state of cells, Mgat 1 enzymes, substrate availability, and genetic factors.^11^, ^33^, ^37^, ^52^

Vitamin D3 supplementation in low‐ and high‐dose arms raised serum 25(OH)D levels from deficient to sufficient levels (>20 ng/mL (50 nM)).^64^, ^65^, ^66^, ^67^ This affirms that patients included in this study were able to successfully supplement and metabolize vitamin D. Nonetheless, our study lacks the power to perform further subgroup analyses, that is, regarding high, mid, and low responders. The EVIDIMS study was aborted after the availability of oral drugs and the increasing trend to self‐medicate with vitamin D made continuation unfeasible. Another limitation is the restriction of analysis to certain time points due to limited source material. For the same reason, we could not address differential glycosylation effects on effector/regulatory subpopulations.

In our study, both treatment arms involved patients who were on stable IFNβ‐1b before study entry, thus eliminating confounding effects due to interactions of IFNβ‐1b with vitamin D. IFNβ is generally known to increase anti‐inflammatory factors while reducing proinflammatory cytokines.^68^ Vitamin D and IFNβ are considered to have synergistic effects in modulating MS.^69^, ^70^, ^71^, ^72^ Combining IFNβ with an analogue of 1,25(OH)2D3 prevented autoimmune encephalitis in animal models of MS.^73^ Additionally, IFNβ enhanced the synthesis of 25(OH)D from sun exposure which was associated with reduced relapse risk.^69^

## Conclusion

We show in an interventional human study that vitamin D affects N‐glycan branching. This is consistent with *ex vivo* and animal studies suggesting that the immunomodulatory effects of vitamin D are associated with regulating N‐glycan branching. We did not observe consistent effects of vitamin D supplementation on immune cell frequencies, which is in support of some but not all previous studies.

## Declaration

Data used in this study are available from the corresponding author upon reasonable request.

## Conflict of Interest

Priscilla Bäcker‐Koduah is a Junior scholar of the Einstein Foundation Berlin. Carmen Duarte‐Infante receives research support from Novartis and Sanofi‐Genzyme and travels support from Novartis. Federico Ivaldi reports no disclosures. Antonio Uccelli has received personal compensation from Novartis, TEVA, Biogen, Merck, Roche, and Genzyme for public speaking and advisory boards. Judith Bellmann‐Strobl received speaking fees and travel grants from Bayer Healthcare, Sanofi Aventis/Genzyme, Biogen and Teva Pharmaceuticals, unrelated to the present scientific work. Klaus‐Dieter Wernecke reports no disclosures. Michael Sy reports no disclosures. Michael Demetriou reports no disclosures. Jan Dörr received research support by Bayer and Novartis, Honoraria for Lectures and Advisory by Bayer, Biogen, Merck Serono, Sanofi‐Genzyme, Novartis, Roche; Travel support by Bayer, Novartis, Merck‐Serono, Biogen. Friedemann Paul served on the scientific advisory boards of Novartis and MedImmune; received travel funding and/or speaker honoraria from Bayer, Novartis, Biogen, Teva, Sanofi‐Aventis/Genzyme, Merck Serono, Alexion, Chugai, MedImmune, and Shire; is an associate editor of Neurology: Neuroimmunology & Neuroinflammation; is an academic editor of PLoS ONE; consulted for Sanofi Genzyme, Biogen, MedImmune, Shire, and Alexion; received research support from Bayer, Novartis, Biogen, Teva, Sanofi‐Aventis/Genzyme, Alexion, and Merck Serono; and received research support from the German Research Council, Werth Stiftung of the City of Cologne, German Ministry of Education and Research, Arthur Arnstein Stiftung Berlin, EU FP7 Framework Pro‐gram, Arthur Arnstein Foundation Berlin, Guthy‐Jackson Charitable Foundation, and NMSS. Alexander Ulrich Brandt is co‐founder and shareholder of Motognosis GmbH and Nocturne GmbH. He is named as an inventor on several patent applications regarding MS serum biomarkers, OCT image analysis and perceptive visual computing.

## Supporting information


**Figure S1.** Serum 25‐hydroxyvitamin D, 25(OH)D levels in the treatment arms over time. Boxplot of serum 25(OH)D levels at each visit in the high‐dose (blue) and low‐dose (gray) arms. Significant differences between both arms for group differences, time change, and interactions were tested using multivariate nonparametric analyses of longitudinal data **P < 0.001. Abbreviations: BL (baseline), V6 (6 months), V12 (12 months), V18 (18 months), (n = 38).

**Figure S2.** Gating strategy for T, B, and NK cells with L‐PHA staining.

**Figure S3.** Heat map correlation matrixes of the MFI of L‐PHA on immune cells with serum 25(OH)D. The figure illustrates the heat map correlation matrixes of the dependence of MFI L‐PHA of immune cells on serum 25(OH)D levels. (A) Baseline, (B) 6 months, (C) 12 and (D) 18 months after supplementation. Each box shows the correlation coefficient between the MFI L‐PHA of immune cells and serum 25(OH)D. The correlations are indicated by the color intensities from blue and from brown to red. Very strong positive correlations are given as 1, whereas strong negative correlations as −1. (n = 38).
