# Supplementary figures and images for: Effect of vitamin D supplementation on N‐glycan branching and cellular immunophenotypes in MS
Source: Ann Clin Transl Neurol. 2020 Aug 23;7(9):1628–41. doi: 10.1002/acn3.51148 (PMC7480923; doi:10.1002/acn3.51148)

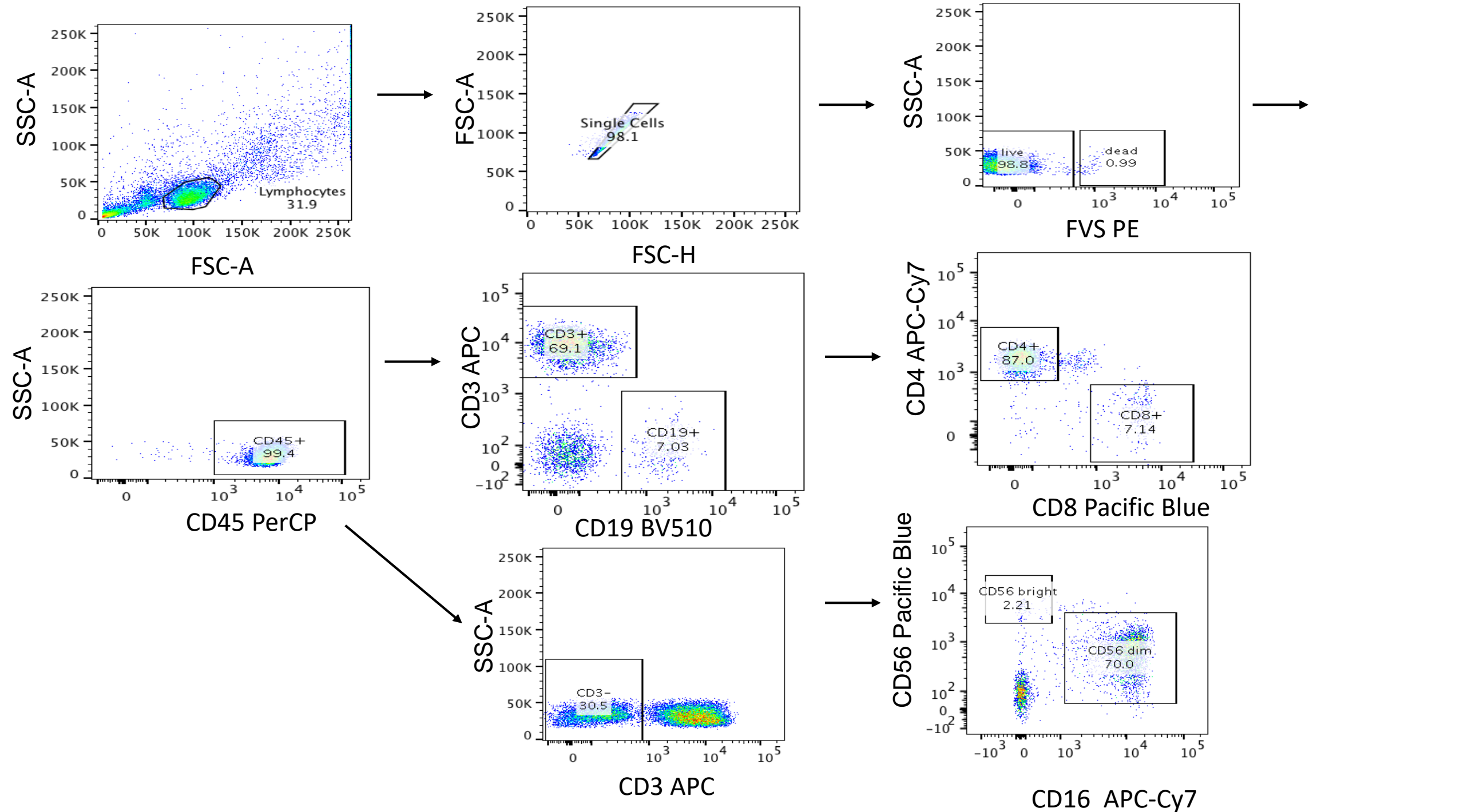

Supplement: Supplementary file 2 — Figure S2. Gating strategy for T, B, and NK cells with L‐PHA staining. [file ACN3-7-1628-s002.pdf]
